# Behavioral and psychological symptoms and hippocampal atrophy in subcortical ischaemic vascular disease

**DOI:** 10.1590/S1980-57642012DN06030011

**Published:** 2012

**Authors:** Chan Tiel, Felipe Kenji Sudo, Carlos Eduardo Oliveira Alves, Gilberto Sousa Alves, Letice Ericeira-Valente, Denise Madeira Moreira, Jerson Laks, Eliasz Engelhardt

**Affiliations:** 1 Instituto de Neurologia Deolindo Couto, Universidade Federal do Rio de Janeiro, Rio de Janeiro RJ, Brazil; 2 Instituto de Psiquiatria, Center for Alzheimer’s Disease (CDA/IPUB), Universidade Federal do Rio de Janeiro, Rio de Janeiro RJ, Brazil; 3 Cognitive and Behavioral Neurology Unit, INDC-CDA/IPUB, Universidade Federal do Rio de Janeiro, Rio de Janeiro RJ, Brazil; 4 Universidade do Estado Rio de Janeiro, Rio de Janeiro RJ, Brazil; 5 Hospital Pró-Cardíaco, Rio de Janeiro, Rio de Janeiro RJ, Brazil

**Keywords:** vascular dementia, executive function, neuropsychology, neuroimaging, cerebrovascular disease, neuropsychiatry

## Abstract

**Background:**

Neuropsychiatric symptoms are common in patients with cognitive impairments, mediated by both neurodegenerative processes and cerebrovascular disease. Previous studies have reported that Behavioral and Psychological Symptoms of Dementia (BPSD) might correlate with severity of cognitive decline. Thus far, the impact of the association between white-matter hyperintensities (WHM) and hippocampal atrophy (HA) on the incidence of these symptoms has been less studied.

**Objective:**

This cross-sectional study aimed to describe the clinical profile of a sample with large extensions of WMH, examining the association between different degrees of HA and cognitive, functional, and behavioral status.

**Methods:**

Fifty outpatients (mean age: 76.86±8.70 years; 58% female; mean schooling: 7.44±4.69 years) with large extensions of WMH (modified-Fazekas scale=3) on MRI and different degrees of hippocampal atrophy (according to de Leon Score) underwent cognitive, functional, and behavioral assessments.

**Results:**

Patients with mild-moderate to severe HA had worse performance on the Mini-Mental State Examination, Cambridge Cognitive Examination, Clinical Dementia Rating and Pfeffer's Functional Activities Questionnaire, compared to the group with none or questionable HA. Appetite/Eating Behavior was the only cluster of neuropsychiatric symptoms associated with presence of HA in Vascular Cognitive Impairment patients.

**Discussion:**

Although HA may exhibit distinct impact on cognitive performance and functional status, it appears to have little effect on behavioral symptoms in patients with high severity WMH.

## Abstract (pt)

**Introdução:**

Sintomas neuropsiquiátricos são comuns em pacientes com déficits cognitivos associados a processos neurodegenerativos e à doença cerebrovascular. Estudos apontaram que os Sintomas Comportamentais e Psicológicos da Demência (SCPD) podem ter relação com a gravidade do declínio cognitivo. Até o presente, o impacto da associação entre hiperintensidades da substância branca (HSB) e atrofia hipocampal (AH) sobre esses sintomas foi pouco explorado na literatura.

**Objetivo:**

Este estudo transversal objetiva descrever as características clínicas de uma amostra com extensas HSB, examinando a influência de diferentes graus de AH sobre os aspectos cognitivos, comportamentais e funcionais.

**Métodos:**

Cinquenta idosos atendidos em ambulatório (idade média: 76.86±8.70anos; 58% mulheres; escolaridade média: 7.44±4.69anos), com extensas HSB (escala modificada de Fazekas=3) e diferentes graus de AH (de acordo com a escala de de Leon) foram submetidos à avaliação cognitiva, funcional e comportamental. Resultados. Pacientes com AH leve-moderada a grave apresentaram piores pontuações no Mini-Exame do Estado Mental, Avaliação Cognitiva de Cambridge, Estadiamento Clínico da Demência e Questionário de Atividades Funcionais de Pfeffer, em comparação com indivíduos com AH ausente ou questionável. Apetite/Comportamento Alimentar foi o único grupo de sintomas neuropsiquiátricos associados à presença de AH em indivíduos com Comprometimento Cognitivo Vascular.

**Discussão:**

Embora a AH pareça apresentar impacto sobre desempenho cognitivo e funcional, parece apresentar pouca influência sobre sintomas comportamentais em pacientes com extensas HSB.

## INTRODUCTION

Vascular cognitive impairment (VCI) can be defined as cognitive alterations due to cerebrovascular lesions. Large and/or small lesions may impair cognition at various degrees of severity, from the mildest to the most severe, constituting a spectrum of VCI/Vascular Dementia (VaD). Concomitant with cognitive impairment, behavioral and psychological symptoms of dementia (BPSD) (neuropsychiatric manifestations) have been reported as frequent features in both vascular and degenerative dementias,^1,2,3^ with an incidence ranging from 80-90% during the course of the disease.^4^ Presence of BPSD has been associated with severity of dementia, poorer outcome and caregiver burden.

In VCI/VaD, impaired cognition has been associated with impaired behavior and rates of behavioral changes have been correlated with rates of cognitive changes.^1,5^ Disruption of specific frontal-subcortical circuits by lacunar infarctions may explain this relationship.^6^ Cortical atrophy might correlate with a distinct profile of behavioral changes. Some authors have reported that VaD, which is more likely to affect subcortical areas, is believed to be involved in mood disorders, especially depression.^27,28^ In contrast, AD, which damages temporal lobe areas, is often associated with psychosis, developing with a higher prevalence of hallucinations and delusions.^1,3,28^ Previous studies^1,3,5,7-9^ have shown that the severity of BPSD was accompanied by clinical worsening of dementia. According to these data, BPSD might help reach a differential diagnosis of dementia subtype.^3,9,28^

Conversely, Steakenborg et al. (2008)^6^ reported an absence of any association between BPSD and presence of HA or WMH in patients with AD. Similarly, Klugman et al. (2009)^29^ assessed patients with AD, and no association between severity of CVD and depression was identified. In these cases, the effect of WMH on mood might have been masked by the pathological sequelae of brain degeneration.

However, other studies have identified differences in the pattern of neuropsychiatric symptoms associated to large-vessel strokes or subcortical disease in VaD:^30,31^ patients with large-vessel VaD can show higher severity of agitation/aggression and euphoria, whereas apathy may be more prevalent in small-vessel VaD.^30^ Depression may be present equally in small-vessel and large-vessel VaD.^31^

Among instruments designed to assess BPSD, the Neuropsychiatric inventory (NPI)^10^ has been widely used, and covers 12 behavioral areas. One study, which evaluated BPSD in VaD and Alzheimer's Disease (AD) patients using NPI, failed to consistently demonstrate behavioral differences between these conditions.^7^ However, in the cited study neither extension of white-matter hyperintensities (WHM) nor hippocampal atrophy (HA) were assessed, and the relationship between brain changes and severity of BPSD could not be established. Therefore, further studies evaluating such symptoms in VCI/VaD and AD are needed, because understanding behavioral patterns associated with neurodegenerative and vascular-related dementias may be useful for clinical practitioners to improve health promotion and clinical management of the diseases.

The aim of the present study was to describe the main neuropsychiatric symptoms in a sample of patients with large extensions of WMH and different degrees of HA on brain MRI. We also examined the impact of HA and WMH on cognitive and functional status in this group of individuals. Our prediction was that there would be little or no effect on frequency and severity of depression, anxiety and apathy in VCI patients associated to the presence of HA. We also hypothesized that psychotic symptoms, global cognitive impairment and functional disability might show higher severity in VCI patients with greater hippocampal volume loss.

## METHODS

**Participants.** This cross-sectional study was conducted with patients who presented vascular subcortical white matter lesions, examined at the Centre for Alzheimer Disease and Related Disorders of the Institute of Psychiatry (CDA/IPUB) and at the Institute of Neurology Deolindo Couto (INDC), Federal University of Rio de Janeiro, between January 2005 and February 2012. Patients with high risk for cerebrovascular disease (CVD), namely those with a Hachinski Ischemic Score^11^ above 7 points, underwent a comprehensive clinical, cognitive, and behavioral assessment by a multidisciplinary team comprising neurologists, psychiatrists, a radiologist and neuropsychologist. Individuals were submitted to brain Magnetic Resonance Imaging (MRI) and only patients with high severity of subcortical white-matter hyperintensities (WMH) were selected. The patients were classified according to the modified Fazekas scale for WMH.^12,13^ Subsequently, these patients were classified into two groups according to hippocampal atrophy using the de Leon score.

Exclusion criteria included history of major psychiatric or neurologic disorders, alcohol or drug abuse, non-corrected visual or auditory disorders, and exposure to neurotoxic substances or cranioencephalic traumatism.

**Neuropsychological, behavioral and functional evaluation.** Cognitive assessments included the Mini-Mental State Examination (MMSE)^14^ and Cambridge Cognitive Examination (CAMCOG).^15^ Clinical stage of cognitive impairment was measured by the Clinical Dementia Rating scale (CDR).^16,17^ Functional status was evaluated by Pfeffer's Functional Activities Questionnaire (FAQ). The 12-item Neuropsychiatric Inventory (NPI) was used to assess behavioral and psychological symptoms (Delusions, Hallucinations, Irritability, Disinhibition, Agitation, Anxiety, Depression, Euphoria, Apathy, Aberrant motor behavior, Night-time behaviors and Appetite and eating behavior.).^10,19^ Both the FAQ and NPI were completed by the patient's caregiver or family member through an interview during the consultation.

**Neuroimaging.** MRI was performed with a 1.5T GE Signa Horizon device. A modified version of the Fazekas scale was applied to measure WMH severity^13^ and those who had a score of 3 (severe extension of WMH) on FLAIR-weighted images were included in this study. Hippocampal Atrophy (HA) was quantified using the de Leon Score.^20,21^ This latter instrument consists of a visual assessment for rating HA scores (no HA=0, questionable=1, mild-moderate=2, and severe=3). de Leon proposed scores 2 and 3 as consistent with AD. Both Fazekas and de Leon scales were visually scored by a trained radiologist and a neurologist (DM and EE), both blind to the clinical and cognitive assessments, and scoring was obtained by consensus. The cases were divided into two groups, one having HA=0-1 ("de Leon 0-1") and the other with HA=2-3 ("de Leon 2-3").

**Statistical analysis.** The Statistical Package for the Social Sciences (SPSS) - version 20 was used for data analysis. The Mann-Whitney Test was used to assess statistically significant differences in age, schooling and performances on cognitive (MMSE and CAMCOG), functional (FAQ) and neuropsychiatric (NPI) evaluations between the "de Leon 0-1" and "de Leon 2-3" groups. Categorical variables (gender and CDR) were analyzed using Pearson's Chi-Square Test for Independence. The level of significance was set at 0.05.

**Ethics.** This study is a branch of a project on Vascular-related cognitive disorders, approved by the Research Ethics Committee of IPUB-UFRJ. Informed consent was obtained from participants or from a family member responsible prior to enrolment.

## RESULTS

A total of 50 elderly outpatients (mean age: 76.86±8.70 years; 58% female; mean schooling: 7.44±4.69 years) with large extensions of WMH (modified-Fazekas scale=3) were studied. Table 1 shows sociodemographic data (age, schooling and gender) of the two groups (de Leon 0-1 and de Leon 2-3). No significant difference between groups was found concerning these data.

**Table 1:** Demographic data.

	de Leon 0-1(m±sd)	de Leon 2-3(m±sd)	p-value
n	22	28	-
Age (years)	74.72±8.58	78.53±8.56	0.80^a^
Schooling (years)	7.68±4.95	7.25±4.57	0.62^a^
Gender (M/F)	8/14	13/15	0.56^b^

a Mann-Whitney test;

b Chi-Square test for independence.

**Figure 1:**
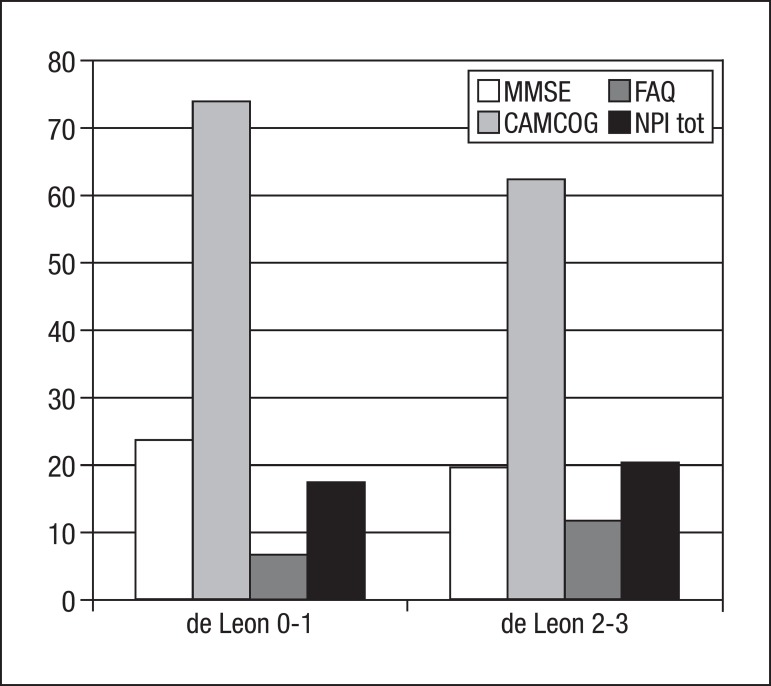
Mean scores on cognitive, functional and behavioral assessments

**Figure 2:**
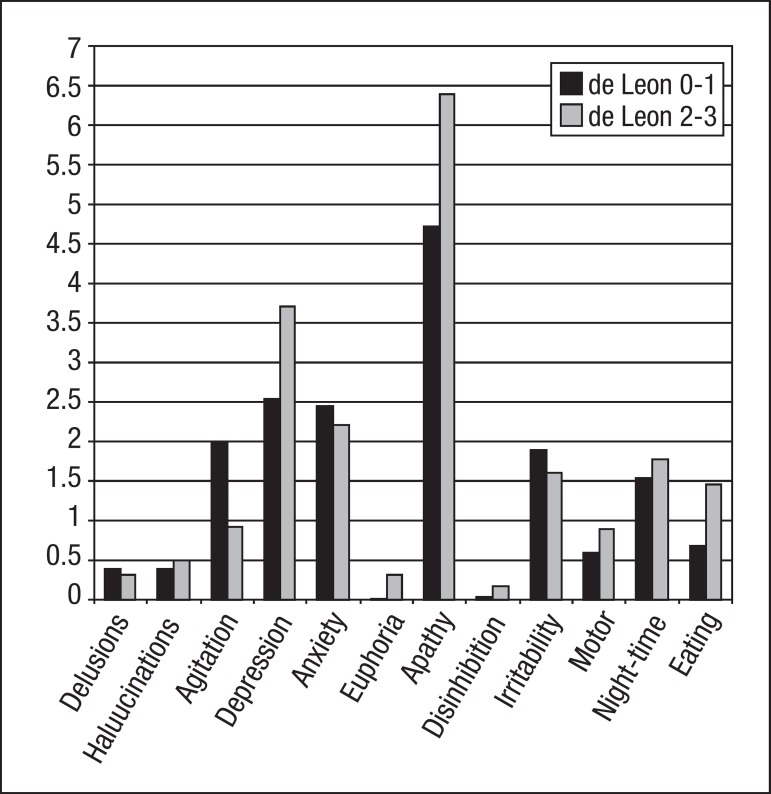
Distribution of mean scores in NPI behavioral areas between de Leon 0-1 and de Leon 2-3 groups.

The comparison between the two HA groups revealed statistical differences on the MMSE, CAMCOG, FAQ and CDR (Table 2). Figure 1 illustrates mean scores on the instruments used to assess cognition, behavioral symptoms and functional status. Among NPI behavioral areas, only changes in Appetite and Eating behavior (p=0.040) showed significant differences, with greater severity observed in the de Leon 2-3 group. Apathy (de Leon 0-1: 4.72±4.60/ de Leon 2-3: 6.42±4.20) and Depression (de Leon 0-1: 2.54±3.26/ de Leon 2-3: 3.71±4.27) were the most prevalent items in both groups, independently of hippocampal score. Despite lacking statistical significance, the values for both were higher in the de Leon 2-3 group. Distribution of scores in NPI behavioral areas is depicted in Figure 2.

**Table 2:** Scores on cognitive, functional and behavioral assessments and statistical differences between groups.

	de Leon 0-1 (m±sd)	de Leon 2-3 (m±sd)	p-value
MMSE	23.59±5.01	19.50±5.02	0.004^a^
CAMCOG	73.54±17.92	62.11±17.43	0.017^a^
FAQ	6.63±9.77	11.67±7.19	0.006^a^
CDR (0/0.5/1/2/3)	7/7/7/1/0	0/4/15/7/2	0.002^b^
Delusions	0.40±1.70	0.32±1.51	0.803^a^
Hallucinations	0.40±1.70	0.50±1.62	0.580^a^
Agitation	2.00±3.84	0.92±2.37	0.478^a^
Depression	2.54±3.26	3.71±4.27	0.454^a^
Anxiety	2.45±3.44	2.21±2.51	1.00^a^
Euphoria	0.0±0	0.32±1.51	0.205^a^
Apathy	4.72±4.60	6.42±4.20	0.175^a^
Disinhibition	0.04±0.21	0.17±0.54	0.406^a^
Irritability	1.90±3.57	1.60±2.92	0.759^a^
Motor behavior	0.59±1.86	0.89±2.02	0.484^a^
Night-time	1.54±2.73	1.78 ±3.27	0.643^a^
Eating	0.68±2.60	1.46±2.48	0.040^a^
NPI total	17.31±17.17	20.25±11.75	0.151^a^

a Mann-Whitney test;

b Chi-Square test for independence; CDR: Clinical Dementia Rate Scale; CAMCOG: Cambridge Cognitive Examination; FAQ: Functional Activities Questionnaire; MMSE: Mini-Mental State Examination.

## DISCUSSION

The aim of the current study was to investigate the effect of the association between large extension of WMH and hippocampal atrophy (HA) on behavioral, cognitive and functional status. Except for higher scores on Appetite / Eating behavior among those subjects with higher HA, there were no statistical differences in NPI total score or in other NPI areas between the two groups (de Leon 0-1 and de Leon 2-3). However, HA may play an important role in cognitive and functional status, as demonstrated by significant performance differences on the MMSE, CAMCOG, CDR and FAQ between groups. Individuals with greater HA scored lower on the MMSE and CAMCOG, and had higher severity of cognitive and functional impairment, as measured by CDR and FAQ, respectively.

In our study, patients with large extensions of WHM (Fazekas=3) had a high frequency and severity of neuropsychiatric symptoms, demonstrated by high mean scores on the NPI, a finding coherent with earlier reports.^1,3,7,22-25^ Patients with extensive areas of WMH might suffer from disconnections in orbitofrontal-subcortical pathways^22,26^ due to cerebrovascular disease or demyelination, which may result in the manifestation of neuropsychiatric symptoms.

Consistent with our findings, Berlow et al. (2010)^36^ evaluated the importance of WMH on behavioral aspects of a sample of patients with AD, showing that WMH, but not HA, were associated with the presence of BPSD. In our study, the main behavioral features observed in both groups were: Apathy, Depression, Anxiety and Irritability. Apathy and Depression were more prevalent in the group with greater HA, while Anxiety and Irritability were more evident in the de Leon 0-1 group. These differences however, did not reach statistical significance. Depression and Apathy were the most prevalent behavioral aspects in our sample, a result that was coherent with our expectations. As mentioned earlier, vascular-mediated disruption in basal ganglia-thalamus-orbitofrontal circuits^7,26-31^ might be associated with these symptoms. On the other hand, studies have suggested that HA may also play a role in Depression and Apathy, due to a direct effect of the hippocampus on these symptoms.^3,9,32,33^ Although Anxiety and Irritability were frequent in the group without significant HA, previous studies have reported that these symptoms might present a greater impact in the later stages of dementia, especially in AD.^3,4,7,9^ Another study showed that Anxiety might be associated to presence of WMH, independently of degree of HA.^36^ Patients included in the de Leon 2-3 group (moderate to severe HA) might present a "mixed" component comprising both vascular and Alzheimer-type neurodegenerative changes, which could be responsible for the divergent neuropsychiatric profile compared with studies in "pure" AD and VCI patients.

Contrary to our initial predictions, patients with higher severity HA did not have greater frequency of Delusions and Hallucinations, in comparison to groups with no or questionable HA. A possible explanation for this might be an overlap between WHM and HA, since presence of vascular-mediated Depression and Apathy may mask others complaints.

Appetite/Eating behavior was the only NPI item that distinguished the groups. Neurodegenerative changes associated to AD may decrease drive to eat by impairing the intrinsic sensation of hunger.^4,9,34^ Weight loss due to abnormal eating behavior has been identified in 40% of patients with AD.^35^ In the same study, the authors associated decreased appetite to hypoperfusion in left orbitofrontal cortex and left anterior cingulate cortex on SPECT scans. Besides degeneration in hippocampus, atrophy in these structures may play a role in appetite regulation.

Limitations of the present study include:

(1) relatively small number of patients;
(2) a heterogeneous sample, including MCI and dementia patients;
(3) some patients might present probable mixed presentations, so substantial overlap of neuropsychiatric symptoms may exist between the two etiologies, vascular and neurodegenerative;
(4) the cross-sectional design, precluding prognostic inferences;
(5) patients were evaluated in a tertiary center, so population-based studies are needed to evaluate the ecological validity of our findings;
(6) although the NPI is a standard tool for measuring BPSD used in various studies, its use may also be viewed as a further limitation of the study, since information may partially reflect caregiver coping styles.

Large prospective studies may provide further insights into the nature of the interaction between cerebrovascular pathology, represented by WMH, and BPSD. Such studies would help to increase our knowledge on the impact of cerebrovascular disease and neurodegenerative disorders on disabling non-cognitive symptoms such as Apathy, Depression and other behavioral problems in cognitively impaired patients.

In patients with subcortical ischaemic vascular disease, hippocampal atrophy affected cognitive performance and functional status, but not most behavioral areas.
